# Causes of preventable death among children of female sex worker mothers in low- and middle-income countries: A community knowledge approach investigation

**DOI:** 10.7189/jogh.14.04052

**Published:** 2024-03-08

**Authors:** Wendy L Macias-Konstantopoulos, Emily Perttu, Swarna Weerasinghe, Duduzile Dlamini, Brian Willis

**Affiliations:** 1 Global Health Promise, Portland, Oregon, USA; 2 Center for Social Justice and Health Equity, Department of Emergency Medicine, Massachusetts General Hospital, Boston, Massachusetts, USA; 3 Harvard Medical School, Boston, Massachusetts, USA; 4 Department of Community Health and Epidemiology, Dalhousie University, Faculty of Medicine, Halifax, Nova Scotia, Canada; 5 Mothers for the Future, Cape Town, Republic of South Africa

## Abstract

**Background:**

Female sex workers (FSW) in low- and middle-income countries (LMIC) are disproportionately vulnerable to poor health, social, and economic outcomes. The children of female sex workers (CFSW) experience health risks based on these challenging circumstances and the unique conditions to which they are exposed. Although country child mortality data exist, little is known about the causes of death among CFSW specifically, thereby severely limiting an effective public health response to the needs of this high-risk group of children.

**Methods:**

The Community Knowledge Approach (CKA) was employed between January and October 2019 to survey a criterion sample of 1280 FSW participants across 24 cities in eight LMIC countries. Participants meeting pre-determined criteria provided detailed reports of deaths among the CFSW within their community of peers. Newborn deaths were gleaned from FSW maternal death reports where the infants also died following birth.

**Results:**

Of the 668 child deaths reported, 589 were included in the analysis. Nutritional deficiencies comprised the leading cause of mortality accounting for 20.7% of deaths, followed closely by accidents (20.0%), particularly house fires, overdoses (19.4%), communicable diseases (18.5%), and homicides (9.8%). Other reported causes of death included neonatal conditions, respiratory illnesses, and suicides.

**Conclusions:**

The causes of CFSW death in these eight countries are preventable with improved protections. Governments, intergovernmental organisations like the United Nations, nongovernmental stakeholder organisations (e.g. sex worker organisations), and funders can implement targeted policies and programmes to protect CFSW and assist vulnerable FSW who are pregnant and raising children. Further research is needed to identify effective child welfare safeguards for CFSW.

Female sex workers (FSW) in low- and middle-income countries (LMIC) disproportionately experience poor health, social, and economic outcomes as compared to the general population [1–8]. FSW are at heightened risk for poor health outcomes related to experiences of physical and sexual violence, food insecurity, barriers to health care, and limited social supports and services [3,5–9]. A recent multi-country study found that leading causes of death among FSW in LMIC are due to maternal causes followed by suicide and homicide [10], highlighting the potential traumatic experiences that also impact the lives of their children [11,12].

A well-established body of literature has demonstrated the health impact of intergenerational trauma. In general, mothers who experience victimisation, either as children or adults, manifest greater maternal depressive symptoms, lower levels of parenting satisfaction, and harsher parenting with higher rates of punitive, threatening, and dissociated behaviours [13–17]. Thus, in the case of the FSW, both their socioecological circumstances and experiences of trauma impact the health of their children.

Children of female sex workers (CFSW) comprise an exceedingly vulnerable group of children resulting from their early and chronic exposure to adversity and toxic stress [18]. Childhood in the unique and onerous environment of commercial sex work can be challenging and child-rearing may be shared among a group of peer FSW [19]. Health risks commonly experienced by CFSW include food insecurity, malnutrition, barriers to health care such as vaccinations and HIV testing (despite HIV-positive mothers), poor caregiver health-seeking behaviours, caregiver neglect, physical and sexual violence, and psychosocial distress due to social marginalisation [20–22].

Although World Health Organization (WHO) country child mortality data exist, little is known about the causes of death among CFSW specifically. One study conducted in Cambodia found that HIV and lung disease were the top leading reported causes of death in CFSW under five years [23]. Mortality data assessments are particularly challenging among populations with poor access to health services. A recent WHO assessment of the status and capacity of health information systems in 133 countries covering 87% of the global population indicated that approximately 40% of global deaths remain unregistered and unaccounted in national health statistics and civil registries [24]. In part due to scarce information technology resources and fragmented healthcare systems, health data gaps and missed deaths were more pronounced in LMIC [20].

Where the lack of robust health and civil registry systems results in underestimating the true burden of mortality [25], alternative scientific methods are needed to accurately collect mortality data among marginalised groups. Methods used vary across settings and include, but are not limited to, household surveys [26], capture-recapture analysis of informant-generated lists of deaths [27], and verbal autopsies [28,29]. By comparison, the Community Knowledge Approach (CKA) offers a low-cost, low-resource, relatively rapid, and validated method for identifying deaths that may otherwise remain unrecorded [23,30,31]. Among low-income communities of Bangladeshi women, the CKA has demonstrated high sensitivity, ranging from 80 to 100%, for identifying neonatal deaths, stillbirths, jaundice-related deaths in ages ≥14 years, and maternal deaths when compared to the house-to-house survey approach [32]. The CKA has also been successfully employed to identify drownings among children one to nine-year-old in a large India study with a sensitivity of 94.5% as compared to the household survey method [33].

The present study, conducted across eight LMIC, uses the CKA method to explore all causes of mortality among CFSW. It is the first study of this magnitude to document causes of death among a particularly marginalised group of children – data essential to understanding differences in causes of death from the general population and to developing targeted prevention strategies.

## METHODS

### Study design and participants

The current study analysed data collected in 2019 as part of a large multi-country cross-sectional cohort study exploring the causes of mortality among FSW and their children. The study employed a modified CKA method to collect information about deaths within primarily urban sex worker social networks in eight LMIC. Study methods have been previously described [10].

Country selection criteria for this study included: large number of FSW, high maternal mortality rate, high HIV infection rates among FSW, partnership with sex worker organisations and local non-governmental organisations, and contribution to the overall geographic regional diversity of the study. Based on these criteria, the eight LMIC selected for this investigation included: Angola, Brazil, Democratic Republic of the Congo (DRC), India, Indonesia, Kenya, Nigeria, and South Africa.

Using criterion sampling, FSW were recruited through local non-governmental (NGO) partners serving FSW to share their knowledge of deaths that occurred within their community of peers during information-gathering group sessions. Participants were recruited at local bars, brothels, parks, and other hotspots. Local partners approached potential study participants and screened interested FSW for study eligibility. Criteria for participation included: (1) age ≥18 years; (2) mother to at least one child aged ≤10 years; (3) engaged in full-time sex work for at least three years; and (4) engaged in peer community activities or living and/or working in communal settings with their peers. Following informed consent, each FSW participated in a single group session, lasting one hour on average, and held in a location deemed safe for FSW by local partners. Participants were not asked to provide any personal identifying data and informed consent was noted with ‘x’ or check mark on the consent form.

### Data collection and quality assurance

Data were collected between 16 January 2019 and 1 October 2019, across 24 cities in the eight study LMIC, by the lead investigator (BW) using an open-ended, semi-structured questionnaire previously used in a similar single LMIC study [23]. Under the direct supervision of the lead investigator, partner-approved local interpreters confirmed accurate forward and backward translation, and were trained on study protocols to assist with data acquisition.

Study participants were asked to share information about any deaths of FSW and CFSW that occurred in the previous five years, since January 2014. Data collected about deceased CFSW included the sex, age at time of death, year of death, and cause of death. Extensive handwritten notes, including verbatim quotes and details surrounding the deaths, were captured for context. Following each group session, the research team reviewed all deaths reported in that location and identified potential duplicates. If any two deaths matched on two reported details, only the details of the first death were recorded.

Data were entered into an Excel database and reviewed by at least two research members for accuracy, completeness, and duplication. Child deaths were coded by three research team members and classified by cause of death as guided by the WHO global classifications of noncommunicable, communicable, and injury-related leading causes of death [34]. Using WHO definitions, deaths related to neonatal conditions were defined as occurring within the first 28 days of life and resulting from birth asphyxia and birth trauma, neonatal sepsis and infections, and preterm birth complications. Age groupings mirrored WHO reporting practices, and 24 years was the maximum age for inclusion in this study representing the age of brain maturation. Although ages ≥18 years fall outside the generally accepted legal definition of a child, the current study focuses on death among children of FSW. Data for the 18–24-year-olds are included given the causes of death among the young adult sons and daughters of FSW are also reflective of the socioeconomic circumstances, health-related social risks, and adverse childhood experiences of CFSW.

### Data analysis

Deaths across all years were combined and organised by country, age group, and cause of death. Cause of death discrepancies were resolved through discussion and review of the original field notes, in conjunction with the lead investigator, until consensus was reached. Descriptive summaries report the total number of child deaths by sex, age group, country, and cause of death.

### Ethics approval

The study protocol, consent forms, and questionnaire were reviewed and approved by the Institutional Ethics Review Board of Portland State University, USA (Protocol #184888). Local partners reviewed the study protocol for local standards and approved the conduction of the study.

## RESULTS

**Figure 1:**
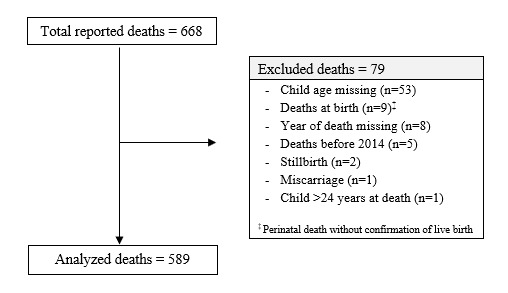
Child death count flow diagram.

A total of 1280 FSW mothers participated in 165 information-gathering sessions across the eight study countries. Of the 668 CFSW deaths reported, 589 were included for analysis (**Figure 1**). The largest number of CFSW deaths were reported in the DRC (n = 272, 46.2%) followed by Kenya (n = 156, 26.5%) and Nigeria (n = 83, 14.1%). More girls (n = 302, 51.3%) than boys were reported among the deceased CSFW. **Table 1** provides death report summaries by country.

**Table 1:** Overview of child death data reports by country

Sites of group sessions			Number of group sessions (cities)	Number of participants, n (%)	Number of deaths included, n (% )	Number of deaths excluded, n (%)	Number of deceased children by sex, n (% total deaths in country)	Age range of the deceased CFSW	Top single leading cause of deaths among CFSW (n, %)
**Country**		**City**							
Angola	Luanda		12	71 (5.5%)	10 (1.7%)	0	Female: 7 (70.0%), male: 3 (30.0%)	1 month to 9 years	Unknown (6, 60.0%)
Brazil	Rio de Janeiro, Salvador, São Paulo		18	80 (6.3%)	10 (1.7%)	0	Female: 1 (10.0%), male: 9 (90.0%)	1 month to 23 years	Homicide (6, 60.0%)
DRC	Bukayu, Kinshasha		27	272 (21.1%)	272 (46.2%)	48 (60.7%)	Female: 150 (55.2%), male: 120 (44.1%), unknown: 2 (0.7%)	2 days to 21 years	Nutritional deficiencies (100, 36.8%)
India	Bangalore, Chennai, Salem, Nashik, Hyderabad, Warangal, Gudibanda		20	152 (11.9%)	19 (3.2%)	0	Female: 6 (31.6%), male: 12 (63.1%), unknown: 1 (5.3%)	6 weeks to 23 years	Suicide (6, 33.3%)
Indonesia	Jakarta		12	76 (5.9%)	7 (1.2%)	1 (1.3%)	Female: 2 (28.6%), male: 4 (57.1%), unknown: 1 (14.3%)	0 days* to 10 years	Communicable diseases (3, 42.9%)
Kenya	Nairobi, Mombasa, Kisumu		18	175 (13.7%)	156 (26.5%)	9 (11.4%)	Female: 72 (46.2%), male: 66 (42.3%), unknown: 18 (11.5%)	1 day to 17 years	Accidents (67, 45.3%)
Nigeria	Lagos, Calabar, Abuja		32	312 (24.4%)	83 (14.1%)	10 (12.7%)	Female: 49 (59.0%), male: 32 (38.6%), unknown: 2 (2.4%)	3 days to 18 years	Overdose (21, 25.3%)
South Africa	Cape Town, Johannesburg, Durban, Port Shepstone		26	144 (11.2%)	32 (5.4%)	11 (13.9%)	Female: 15 (46.9%), male: 17 (53.1%)	1 week to 21 years	Homicide (9, 28.1%)
TOTAL	24		165	1,280	589	79	Female: 302 (51.3%), male: 263 (44.6%), unknown: 24 (4.1%)	0 days* to 23 years	

CFSW – children of female sex workers

*0 days indicates death occurred within the first 24 hours following birth.

### Causes of child death by country

**Figure 2:**
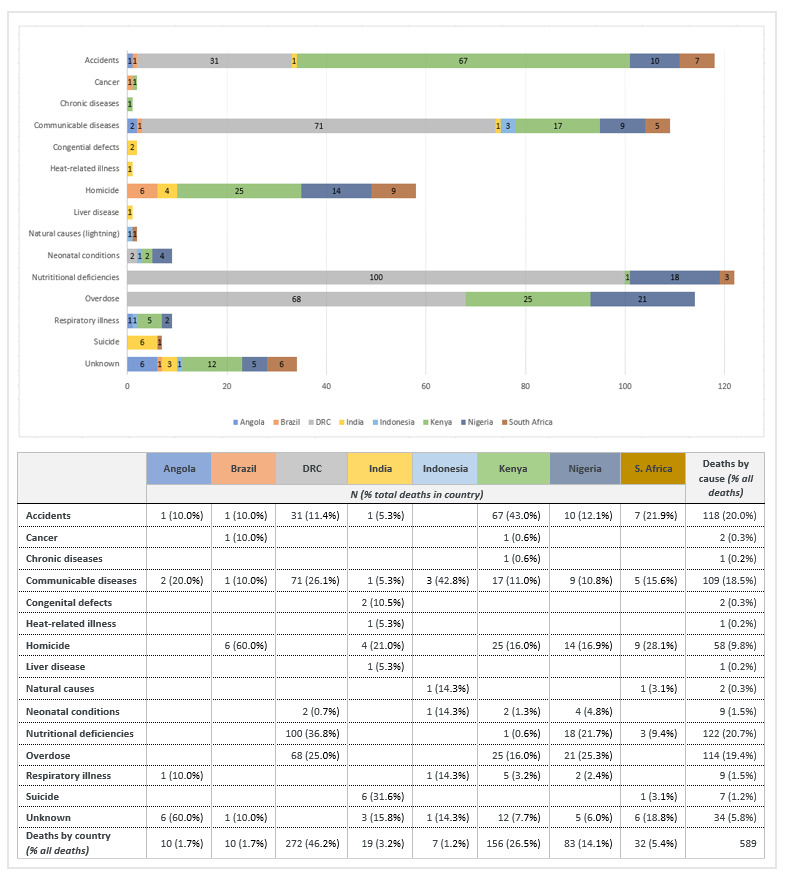
Causes of child death by country.

Country-specific causes of CFSW deaths varied. In aggregate, the leading causes of death were nutritional deficiencies (n = 122, 20.7%), accidents (n = 118, 20%), overdoses (n = 114, 19.4%), communicable diseases (n = 109, 18.5%), and homicides (n = 58, 9.8%), accounting for 88.4% of deaths. Fewer deaths secondary to neonatal conditions, respiratory illnesses, suicides, natural causes (lightning strikes), cancer, and congenital defects were reported. **Figure 2** provides detailed causes of death by country.

#### Nutritional deficiencies

Of the 122 deaths due to nutritional deficiencies, kwashiorkor accounted for 81.1% of deaths and was reported only in the DRC. The other 23 deaths reported were due to malnutrition resulting from a lack of food and occurred in Nigeria, South Africa, Kenya, and the DRC.

#### Accidents

The 118 fatal accidents reported were both related to life-threatening incidents and unsafe actions or omissions (i.e. neglect). Mechanisms of accidental death included house fires (55.9%), road accidents (24.6%), and other causes (19.5%) involving suffocation, choking, drowning, roof collapses, stampede trampling, and falls from heights.

Most of the house fire deaths were reported in Kenya (68.2%) followed by the DRC (12.1%), South Africa (10.6%), Nigeria (7.6%), and Angola (1.5%). Details collected suggested that deaths related to house fires were due to sleeping children being left home alone, behind locked doors, with kerosene oil lamps or candles burning, while their mothers worked the night shift. Suffocations occurred among infants due to mothers rolling onto them while sleeping or intoxicated. On the other hand, deaths resulting from a choking incident were commonly due to rushed feeding sessions, overfeeding by a young sibling, and being fed – or being left unattended immediately after a feeding – in a position of increased risk for aspiration.

#### Overdoses

Despite overdoses, participants reported that medicating children to induce sleep is common practice among FSW mothers who lack the childcare needed to work. Substances identified in fatal overdoses included diazepam (59.6%, ‘Valium’), chlorphenamine (16.7%, ‘Piriton’), tramadol (5.3%), alcohol (5.3%), unknown (3.5%), codeine (3.5%), ‘sleeping medication’ (1.8%), opium (1.8%), ‘cough/cold medicine’ (1.8%), and flunitrazepam (0.9%, ‘Rohypnol’).

#### Communicable diseases

Of the 109 deaths due to communicable diseases, the largest proportion was reported in the DRC (65.1%) including cases of malaria (91.6%), HIV (5.6%), and tuberculosis (2.8%). In Kenya, 17 (15.6%) deaths due to communicable diseases included cases of HIV (76.5%), malaria (11.8%), measles (5.9%), and meningitis (5.9%).

#### Homicide

The largest number of homicides were reported in Kenya (43.1%) followed by Nigeria (24.1%), South Africa (15.3%), Brazil (10.3%), and India (5.2%). While homicides in Brazil involved firearms and were associated with criminality, mechanisms of homicide in the other countries included strangulation and physical abuse by the mother or clients of the mother, filicide-suicide, violent sexual assaults, public stoning or burning as punishment for wrongdoings (‘mob murder’), and poisonings.

### Ages of the deceased children

**Figure 3:**
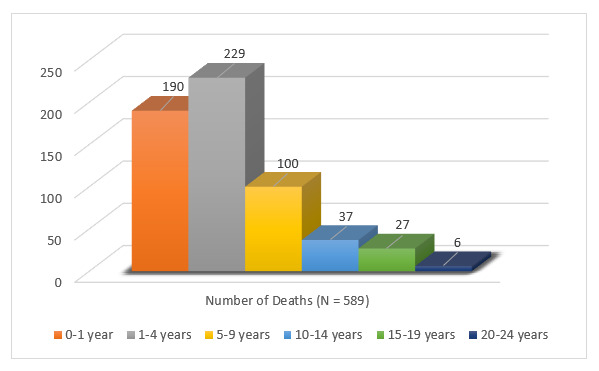
Children of female sex workers (FSW) death count by age group.

Age at time of death ranged from <1 day (newborn death within 24 hours of birth) to 23 years. The highest death count was reported among children aged one to four years (n = 229, 38.9%) followed by infants under the age of one year (n = 190, 32.3%) and five to nine-year-olds (n = 100, 17%) (**Figure 3**).

### Causes of child death by age group

**Figure 4:**
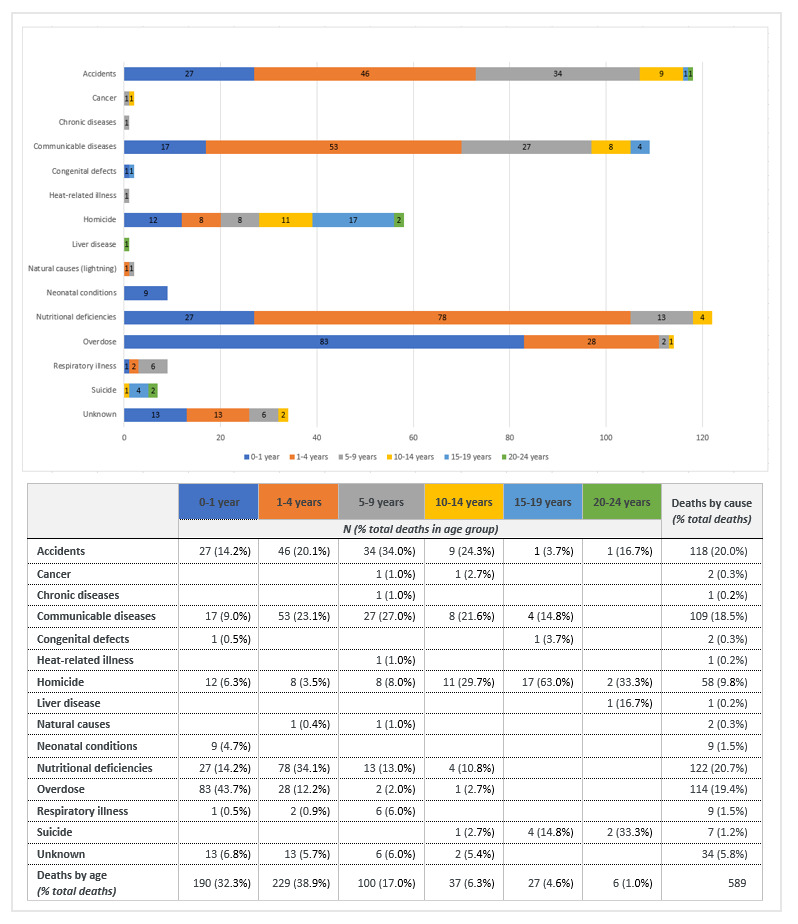
Causes of child death by age.

Leading causes of child death varied by age group (**Figure 4**). Among infants, overdoses comprised the leading cause of death (43.7%) followed equally by accidents and nutritional deficiencies (14.2% each), and communicable diseases (8.9%). Among children one to four years old, nutritional deficiencies (34.1%), communicable diseases (23.1%), and accidents (20.1%) were the top three leading causes of death. The increasingly independent five to nine-year-olds were more likely to die from accidents (34%) although communicable diseases (27%) continued to impact this age group. Homicide was the leading cause of death among 15 to 19-year-old CFSW (63%), whereas homicide and suicide equally impacted the 20 to 24-year-olds.

### Causes of death by country and age group

In descending order, reported deaths were most numerous among the early age groups of one to four-year-olds, infants, and five to nine-year-olds. The largest proportion of CFSW deaths (n = 511, 86.8%) were reported in three sub-Saharan African countries – the DRC (n = 272), Kenya (n = 156), and Nigeria (n = 83). In the DRC, 58.6% of infant deaths were due to overdoses, 52.1% of the deaths reported among one to four-year-old children were due to nutritional deficiencies, and 53.6% of deaths in the five to nine-year-old age group were attributed to communicable diseases. In Kenya, accidents comprised the leading cause of death across all three early age groups (34% infants, 54.7% one to four-year-olds, and 48.6% five to nine-year-olds). Overdose was the second leading cause of death among Kenyan infants (32.1%) and the leading cause of death among Nigerian infants (55.6%). Homicide was a cause of death among 15 to 19-year-olds in Brazil, India, Kenya, Nigeria, and South Africa. **Table 2** cross-references country and age data to determine the causes of death by age group and country.

**Table 2:** Causes of death by country and age

Country	0–1 year (n, %)	1–4 years (n, %)	5–9 years (n, %)	10–14 years (n, %)	15–19 years (n, %)	20–24 years (n, %)	Total, n
Angola	5: unknown (4, 80%), Comm disease (1, 20%)	2: unknown (2, 100%)	3: accidents (1, 33%), comm disease (1, 33%), respiratory illness (1, 33%)	0	0	0	10
Brazil	2: accidents (1, 50%), unknown (1, 50%)	0	0	1: cancer (1, 100%)	6: homicide (5, 83%), comm disease (1, 17%)	1: homicide (1, 100%)	10
DRC	87: overdose (51, 59%), nutritional def (21, 24%), Comm disease (8, 9%), accidents (5, 6%), neonatal condition (2, 2%)	144: nutritional def (75, 52%), comm disease (41, 29%), overdose (15, 10%), accidents (13, 9%)	28: comm disease (15, 54%), accidents (8, 28%), nutritional def (3, 11%), overdose (2, 7%)	9: comm disease (5, 56%), accidents (3, 33%), nutritional def (1, 11%)	3: comm disease (2, 67%), accidents (1, 33%)	1: accidents (1, 100%)	272
India	2: congenital defects (1, 50%), unknown (1, 50%)	2: homicide (2, 100%)	4: unknown (2, 50%), accident (1, 25%), heat-related illness (1, 25%)	2: comm disease (1, 50%), suicide (1, 50%)	7: suicide (4, 57%), homicide (2, 29%), congenital defects (1, 14%)	2: liver disease (1, 50%), suicide (1, 50%)	19
Indonesia	2: comm disease (1, 50%), neonatal conditions (1, 50%)	2: comm disease (1, 50%), respiratory illness (1, 50%)	2: comm disease (1, 50%), natural cause (1, 50%)	1: unknown (1, 100%)	0	0	7
Kenya	53: accidents (18, 34%), overdose (17, 32%), homicide (9, 17%), unknown (4, 7%), comm disease (3, 6%), neonatal conditions (1, 2%), nutritional def (1, 2%)	53: accidents (29, 55%), overdose (8, 15%), unknown (7, 13%), comm disease (5, 9%), homicide (3, 6%), respiratory illness (1, 2%)	35: accidents (17, 48%), comm disease (7, 20%), respiratory illness (4, 11%), homicide (3, 9%), unknown (2, 6%), cancer (1, 3%), chronic disease (1, 3%)	9: homicide (4, 45%), accidents (3, 33%), comm disease (2, 22%)	6: homicide (6, 100%)	0	156
Nigeria	27: overdose (15, 56%), neonatal conditions (4, 15%), nutritional def (3, 11%), homicide (2, 7%), accidents (1, 3·7%), respiratory illness (1, 3·7%), unknown (1, 3·7%)	20: comm disease (6, 30%), overdose (5, 25%), accidents (4, 20%), nutritional def (2, 10%), unknown (2, 10%), homicide (1, 5%)	20: nutritional def (10, 50%), accidents (3, 15%), homicide (3, 15%), comm disease (2, 10%), respiratory illness (1, 5%), unknown (1, 5%)	12: homicide (5, 42%), nutritional def (3, 25%), accidents (2, 17%), overdose (1, 8%), unknown (1, 8%)	4: homicide (3, 75%), comm disease (1, 25%)	0	83
South Africa	12: comm disease (4, 33%), unknown (3, 25%). accidents (2, 17%), nutritional def (2, 17%), homicide (1, 8%)	6: homicide (2, 33%), unknown (2, 33%), natural cause (1, 17%), nutritional def (1, 17%)	8: accidents (4, 50%), homicide (2, 25%), comm disease (1, 12·5%), unknown (1, 12·5%)	3: homicide (2, 67%), accidents (1, 33%)	1: homicide (1, 100%)	2: homicide (1, 50%), suicide (1, 50%)	32
Total	190	229	100	37	27	6	589

Comm disease – communicable disease, nutritional def – nutritional deficiencies

## DISCUSSION

CFSW comprise a socially marginalised group of children whose causes of death may differ from the general population. Identifying causes of death among CFSW is essential for developing targeted strategies and prevention programs. The current multi-country study is the first to offer unique insight into the causes of death among CFSW in LMIC.

WHO Africa has acknowledged the existence of gaps in child mortality data across subgroups within countries [35], complicating efforts to achieve Millennium Development Goal 4 (two-thirds global reduction in under-5 child mortality) [36] and Sustainable Development Goal 3 (ensuring healthy lives and promoting wellbeing for all ages) [37]. An equitable approach to child mortality reduction requires additional efforts to identify and understand differences in causes of death across subgroups with varying levels of social vulnerability. The present study highlights potential differences in causes of death among CFSW as compared to WHO general child mortality country data [34].

According to WHO 2019 data, neonatal conditions and respiratory illness comprise the top two leading causes of death among the general infant population in the DRC, while communicable diseases (malaria, measles) were the leading causes of death among one to four-year-old children [34]. In the current study, infant CFSW in the DRC died predominantly from accidental overdoses with sedating substances administered to induce sleep and the leading cause of one to four-year-old CFSW death in the DRC was related to nutritional deficiencies from lack of access to food, possibly resulting from abject poverty associated with the economic situation of many FSW. Consistent with WHO country data, communicable diseases were responsible for CFSW deaths from infancy to 19 years of age in the DRC.

In Kenya, WHO 2019 mortality data identified neonatal conditions as the leading cause of death among infants. Following infancy, communicable diseases were in the top three causes of death among one to 24-year-olds while accident-related deaths were due to road injuries (ranked in the top three causes of death across the five to 24-year-old age groups) and drownings (ranked seventh among one to four-year-olds) [34]. Among CFSW in Kenya, the current study found that accidents comprised the leading cause of death from infancy through nine years of age with unattended children in house fires playing a prominent role.

Homicide deaths among CFSW are worth noting. In the current study, homicides comprised the third leading cause of death among Kenyan infants and were all the result of filicide by their FSW mothers, accounting for 17% of all Kenyan infant deaths. In the WHO 2019 data, interpersonal violence does not appear in the top 10 causes of death among the general Kenyan population until the 15 to 19-year-old age group where it ranks seventh on the list [34]. Homicide also accounted for a progressively increasing number of CFSW death in Kenya responsible for 5.7% deaths among one to four-year-olds, 8.6% among five to nine-year-olds, 44% among 10 to 14-year-olds, and 100% among 15 to 19-year-olds. The same pattern is evident in Nigeria where WHO 2019 first identifies interpersonal violence as a leading cause of death among 15 to 19-year-olds, while among CFSW in the current study, homicide is a prominent cause of death across all the age groups (infants = 7.4%; one to 5 years = 5%; five to nine years = 15%; 10 to 14 years = 41.7%, and 15 to 19 years = 75%) [34]. Anecdotally, Nigerian participants reported that sons of FSW frequently leave their homes at a young age and live on the streets where they are exposed to gang-related violence.

Finally, nutritional deficiency deaths were most frequently reported in the DRC where they account for more than a third of all deaths among infants and under five-year-olds. These findings are particularly concerning given global supply chain and climate-related food ecology challenges threaten to further exacerbate food insecurity among FSW mothers and their children.

With an estimated 40% of global deaths left unrecorded [24], the incomplete status and limited capacity of health information systems as assessed by the WHO should prompt regionally coordinated, inter-governmental mitigation strategies. Within LMIC, government agencies must partner across districts to reinforce access to information technology resources, build centralised data systems with information-sharing capabilities, and encourage more consistent and accurate recording of every birth and death regardless of whether the healthcare system was accessed. Concerted efforts to support more robust data capture in national health statistics and civil registries should involve special provisions undertaken to account for the unique challenges in health data collection among hard-to-reach populations. Improved morbidity and mortality data among subgroups with high indices of social vulnerability would allow for a better understanding of health inequities and the targeted interventions needed to close the gaps.

### Strengths and limitations

The CKA is a low-cost, low-resource, relatively rapid, and validated method for identifying causes of death among communities of adult women and children in LMIC [32,33]. Although the current study does not include an internal validity measure, the methodology borrows from previous studies demonstrating that women in a community are able to accurately report the deaths of children and other women in their community. Household survey verification of these child deaths would be challenging as this would require that households of origin be aware of a female family member’s status as a FSW mother, and knowledgeable of the circumstances surrounding the child’s death. Similarly, triangulation with official records would require deceased children to be identified as CFSW in civil death registries or health facility records. The possibility of selection bias should be noted given that there may be some fundamental differences between FSW who opt to participate versus decline to participate in the study.

Further, time constraints made it necessary to adjourn group sessions before fully exhausting the memory of participants. Additionally, the inherent differences between study locations in each country (e.g. average population densities) and the inability to collect data in more than an average of three cities per country, limit the extent to which these data are generalisable and representative of all CFSW deaths in any given country. Finally, without accurate size estimates of the total CFSW population in any given country, mortality rates cannot be estimated. Therefore, while these data are useful for identifying local outcomes and guiding targeted public health interventions, they cannot be used to estimate mortality rates among CFSW in any given country or draw conclusions about differences in causes of CFSW death across countries.

## CONCLUSIONS

The current study begins to address a crucial and concerning knowledge gap on the causes of death among CFSW. These findings not only highlight the need for focused data collection efforts on marginalised, hard-to-reach subgroups, but they also call for a multifaceted approach to mitigating harm and preventing death. Developing effective, low-cost methods to account for missed deaths, particularly among vulnerable populations in LMIC, is a critical step toward more accurate mortality assessments. The children of FSW experience significant health risks and should be prioritised for morbidity and mortality assessments to guide appropriate prevention strategies and adequate allocation of resources. With adequate data, governments, intergovernmental organisations like the United Nations, non-governmental organisations, and funders can implement targeted policies and programmes to protect CFSW and assist vulnerable pregnant and parenting FSW mothers. More research is needed to determine if policies and programmes that aim to reduce stigma, increase access to health services, enhance parenting skills, and provide social supports (e.g. childcare) are effective harm-reduction strategies worth pursuing.
